# Overexpression of E3 Ubiquitin Ligase Gene *AdBiL* Contributes to Resistance against Chilling Stress and Leaf Mold Disease in Tomato

**DOI:** 10.3389/fpls.2017.01109

**Published:** 2017-06-30

**Authors:** Shuangchen Chen, Hongjiao Zhao, Mengmeng Wang, Jidi Li, Zhonghong Wang, Fenghua Wang, Airong Liu, Golam J. Ahammed

**Affiliations:** ^1^College of Forestry, Henan University of Science and TechnologyLuoyang, China; ^2^Department of Plant Science, Tibet Agriculture and Animal Husbandry CollegeLinzhi, China; ^3^Department of Horticulture, Zhejiang UniversityHangzhou, China

**Keywords:** E3 ubiquitin ligase gene, redox signal, reactive oxygen species (ROS), reactive nitrogen species (RNS), *S*-nitrosylation, transgenic tomato

## Abstract

Ubiquitination is a common regulatory mechanism, playing a critical role in diverse cellular and developmental processes in eukaryotes. However, a few reports on the functional correlation between E3 ubiquitin ligases and reactive oxygen species (ROS) or reactive nitrogen species (RNS) metabolism in response to stress are currently available in plants. In the present study, the E3 ubiquitin ligase gene *AdBiL* (Adi3 Binding E3 Ligase) was introduced into tomato line Ailsa Craig via *Agrobacterium*-mediated method. Transgenic lines were confirmed for integration into the tomato genome using PCR. Transcription of *AdBiL* in various transgenic lines was determined using real-time PCR. Evaluation of stress tolerance showed that T_1_ generation of transgenic tomato lines showed only mild symptoms of chilling injury as evident by higher biomass accumulation and chlorophyll content than those of non-transformed plants. Compared with wild-type plants, the contents of AsA, AsA/DHA, GSH and the activity of GaILDH, γ-GCS and GSNOR were increased, while H_2_O_2_, $O_{2}^{.-}$, MDA, NO, SNOs, and GSNO accumulations were significantly decreased in *AdBiL* overexpressing plants in response to chilling stress. Furthermore, transgenic tomato plants overexpressing *AdBiL* showed higher activities of enzymes such as G6PDH, 6PGDH, NADP-ICDH, and NADP-ME involved in pentose phosphate pathway (PPP). The transgenic tomato plants also exhibited an enhanced tolerance against the necrotrophic fungus *Cladosporium fulvum*. Tyrosine nitration protein was activated in the plants infected with leaf mold disease, while the inhibition could be recovered in *AdBiL* gene overexpressing lines. Taken together, our results revealed a possible physiological role of *AdBiL* in the activation of the key enzymes of AsA–GSH cycle, PPP and down-regulation of GSNO reductase, thereby reducing oxidative and nitrosative stress in plants. This study demonstrates an optimized transgenic strategy using *AdBiL* gene for crop improvement against biotic and abiotic stress factors.

## Introduction

Ubiquitination is a common regulatory mechanism that occurs in all eukaryotes to regulate diverse cellular and developmental processes at post-translational level (Liu et al., 2013). In plants, cellular processes that are controlled by ubiquitination are quite diverse, such as, cell cycle, differentiation, hormone responses, protein trafficking, and responses to environmental stresses, and thus signifying a broader functional aspect of ubiquitination. Ubiquitination involves an enzymatic cascade that includes Ub-activating (E1), Ub-conjugating (E2), and Ub-ligase (E3) enzymes. E3 proteins are normally classified into four main subfamilies depending on their structure and mode of action: HECT (Homologous to E6-associated protein C-Terminus), RING (Really Interesting New Gene), U-Box and cullin-RING ligases (Fang et al., 2015).

In plants, the E3s are encoded by a large number of genes. For example, number of genes encoding E3s is more than 1,200 in *Arabidopsis*. Such a high number of genes relative to the other eukaryotes underline the importance of E3s in regulating plant processes. Many studies have demonstrated that E3s are key regulators in the response of plants to abiotic stress, hormone signaling, photomorphogenesis, cell cycling and plant–microbe interactions (Hervé et al., 2011; Ling and Jarvis, 2015). *AtCHIP*, a U-box-containing E3 ubiquitin ligase, plays a critical role in temperature stress tolerance in *Arabidopsis*. Overexpression of *AtCHIP* in *Arabidopsis* renders plants more sensitive to both low- and high-temperature stresses. For instance, an increased electrolyte leakage was observed in leaves of *AtCHIP* overexpressing plants in response to chilling (Yan et al., 2013). *Arabidopsis* RING E3 *AtAIRP3/LOG2* is a positive regulator of the ABA-mediated drought and salt stress tolerance (Kim and Kim, 2013). Liu et al. (2013) demonstrate that RING-finger protein from *Zea mays* (*ZmRFP1*) confers drought stress tolerance to transgenic tobacco not only by increasing the ability to retain water, but also by reducing reactive oxygen species (ROS) accumulation and membrane damage through activation of the antioxidant system. *Arabidopsis* plants lacking ubiquitin E3 ligase Suppressor of Ppi1 Locus1 (*SP1*) are hypersensitive to salt, osmotic, and oxidative stresses, whereas plants overexpressing *SP1* are considerably more stress tolerant than wild-type (Ling and Jarvis, 2015). However, *AtATL80*, a PM-localized ATL-type RING E3 Ub ligase, participates in the Pi mobilization and cold stress response as a negative factor in *Arabidopsis. AtATL80*-overexpressors are significantly more sensitive to cold stress than wild-type plants, while the *AtATL80* mutant lines exhibit an increased tolerance to cold stress (Suh and Kim, 2015).

On the other hand, limited information is available about the roles of RING-finger proteins in plant defense. Overexpression of the *CaRFP1* gene in the transgenic *Arabidopsis* plants increases disease susceptibility to *Pseudomonas syringae* pv. tomato infection, accompanied by reduced *PR-2* and PR-5 gene expression, suggesting that the *CaRFP1* acts as an E3 ligase for polyubiquitination of target PR proteins (Hong et al., 2007). Yu et al. (2013) demonstrated that EIRP1 E3 ligase positively regulates plant powdery mildew resistance by mediating proteolysis of the negative regulator *VpWRKY11* through degradation by the 26S proteasome. Overexpression of NITROGEN LIMITATION ADAPTATION (*NLA*) that encodes an ubiquitin E3 ligase enzyme results in a reduction in plant susceptibility to *Heterodera schachtii* (Hewezi et al., 2016). In spite of the progress made in understanding molecular and biological function of E3 Ub ligases in model plant *Arabidopsis*, few studies have attempted to elucidate the molecular and functional aspects of the RING-type E3 ligases of vegetable crops such as tomato.

Tomato (*Solanum lycopersicum* L.) is a warm season vegetable that prefers relatively warm weather. An air temperature of 10°C or below delays seed germination, inhibits vegetative development, reduces fruit set, and impairs fruit ripening in tomato (Haghighi et al., 2014). Low molecular weight antioxidants, such as ascorbate, glutathione, and tocopherol, are informative redox buffers that interact with numerous cellular components. ROS and reactive nitrogen species (RNS), especially H_2_O_2_ and NO, respectively, can modulate signaling networks that control growth, development and stress response both independently and synergistically (Miller et al., 2010; Kolupaev et al., 2015; Poor et al., 2015). *Cladosporium fulvum* is a potentially serious fungal pathogen to tomato production which has attracted attention as a model system in fungal phytopathology (de Wit, 2016). Twelve races of *C. fulvum* with a different virulence spectrum have been identified (Iida et al., 2015). On the other hand, new *C. fulvum* races have been evolved that can overcome introduced *Cf* resistance genes by selection pressure imposed by *Cf* genes. At present, pesticide application is the main method to prevent and control tomato leaf mold disease, but the excessive use of chemical fungicides is blamed for polluting water and causing health problems. It has been reported that overexpression of tomato 13-Lipoxygenase gene *TomloxD* enhances tolerance to *C. fulvum* and high temperature stress by regulating endogenous jasmonic acid synthesis (Hu et al., 2013). However, there are only a few reports on how E3 ubiquitin ligase affects redox homeostasis and antioxidant signaling associated with ROS and RNS accumulation in response to chilling stress and *C. fulvum* infection in tomato.

To obtain a better understanding of the function of plant E3 ubiquitin ligase in response to abiotic stresses, a functional analysis of tomato E3 ubiquitin ligase *AdBiL* gene in transgenic tomato plants was performed. The transgenic tomato plants conferred significantly improved tolerance to chilling stress and *C. fulvum*. Further investigation revealed that tyrosine nitration protein mediated by GSH and *S*-nitrosogluthathione reductase (GSNOR) was actively involved in *AdBiL*-mediated tolerance to chilling stress, by maintaining a lower H_2_O_2_ and RNS accumulation in the leaves of tomato.

## Materials and Methods

### Plant and Strain Materials

Tomato (*S. lycopersicum* L.) cv. Ailsa Craig was used for transformation and physiological analysis in this work which is sensitive to chilling stress and *C. fulvum* (Hu et al., 2013; Ren et al., 2014). Tomato seeds were surface-sterilized with 75% ethanol for 20 s and 8% sodium hypochlorite for 12 min, subsequently rinsed several times with sterile distilled water. These seeds were sown on phytohormone-free 1/2 MS medium containing 15 g L^-1^ sucrose and 8 g L^-1^ agar. The surface sterilized seeds were germinated in a culture chamber at 25°C, 16/8-h (light/dark) photoperiod and light intensity of 50–60 μmol m^-2^ s^-1^. Cotyledons were excised about 10 days after sowing.

### Construction of *AdBiL* Expression Cassette and Plant Transformation

*AdBiL* cDNA was generated by RT-PCR from total RNA of tomato seedlings (cv. Ailsa Craig). We used the primer pair *AdBiL* -F (5′-CTGCATCATCTGCTGCTCAT-3′) and *AdBiL* -R (5′-GCTCGGCTACCACTATCA-3′) to amplify the original *AdBiL* cDNA (XM_015227365.1). The amplified fragments of the original *AdBiL* cDNA were cloned into the pCR2.1 TOPO vector (Invitrogen, United States). The *AdBiL* cDNA clone was digested with *Xba* I and *Bam*H I restriction enzymes, and then excised fragment was ligated to corresponding site of the pBI121 expression vector under the control of CaMV 35S promoter and nopaline synthase (nos) terminator, hygromycin phosphotransferase gene (*hpt* II) for plant selection, and neomycin phosphotransferase gene (*npt* II) for bacterial selection.

The resulting expression plasmid was introduced into *Agrobacterium tumefaciens* (strain EHA105) using the freeze-thaw method, which was subsequently used for stable transformation of tomato. Regenerated plants (T_0_) on culture medium containing 100 mg L^-1^ kanamycin and cefotaxime sodium (250 mg L^-1^) were screened by PCR using the *LeRma1*-specific primer pair. The transgenic plants were examined by genomic PCR, Southern blot and real-time PCR analysis. Transgenic plants containing *AdBiL* gene were transferred to a greenhouse and grown to maturity. T_1_ seeds from transgenic tomato plants expressing *AdBiL* were used for molecular analyses and further physiological experiments.

### Molecular Analyses of Transgenic Plants

Total genomic DNA was isolated from young leaves of the wild-type plant and putative transgenic plants (T_1_). The expected gene fragment in transformants was amplified using the following primer pairs: forward (35S): 5′-GACGCACAATCCCACTATCC-3′ and reverse (*AdBiL*): 5′-CCTGCTCCAATGTTAG-3′. PCR reaction mixture (25 μL) consisting of 10× PCR reaction buffer, 50 ng templates DNA, 0.2 mmol L^-1^ deoxynucleotide triphosphates, 1.5 mmol L^-1^ MgCl_2_, 0.2 μmol L^-1^ of each primer and 1 unit of Taq DNA polymerase. After the initial denaturing for 2 min at 94°C, PCR was performed during 35 cycles (denaturing at 94°C for 45 s, annealing at 56°C for 1 min, synthesis at 72°C for 1.5 min). Thereafter, the program was terminated by an extension at 72°C for 10 min. The amplification was analyzed by electrophoresis in 1% agarose-ethidium bromide gels.

### Determination of Transcript Abundance

Total RNA was isolated from tomato young leaves in different treatments using Trizol reagent (Sangon, China) according to the manufacturer’s instruction. Genomic DNA was removed with RNeasy Mini Kit (Qiagen, Germany). Total RNA (1 μg) was reverse-transcribed using ReverTra Ace qPCR RT Kit (Toyobo, Japan) following the manufacturer’s instruction. Quantitative real-time PCR was performed using the iCycler iQ^TM^ real-time PCR detection system (Bio-Rad, Hercules, CA, United States) using gene-specific primers 5′-TGACCGTGTTCAAGCTCTTC-3′ (forward) and 5′-GGATCGTCGAGTAGCAGACA-3′ (reverse). Each reaction (25 μL) consists of 12.5 μL SYBR Green PCR Master Mix (Takara, Japan), 1 μL of diluted cDNA and 0.1 μmol of forward and reserve primers. PCR cycling conditions were as follows: 95°C for 3 min and 40 cycles of 95°C for 10 s 58°C for 45 s. *Actin* of tomato (GenBank U60480) was used as an internal standard and was amplified with primers 5′-TGGTCGGAATGGGACAGAAG-3′ (forward) and 5′-CTCAGTCAGGAGAACAGGGT-3′ (reverse). The quantification of mRNA levels is based on the method of Livak and Schmittgen (2001).

### Biomass and Chlorophyll Content Analysis

Nine plants from transgenic lines (M_3_, M_6_, and M_12_) and control were randomly selected and divided into shoots and roots. Fresh weight of the selected plants was measured. Then they were dried in an oven at 80°C for 24 h and weighed to record their dry weights, respectively. Chlorophyll was extracted from the third fully developed leaves and the contents were determined by using a spectrophotometer (UV-1800, shimadzu, Japan) as described by Goodwin (1965).

### Evaluation of Stress Tolerance

T_1_ seeds from transgenic tomato plants were used for chilling tolerance assay. Seeds of cv. Ailsa Craig were also used as a control. One half of untransformed and T_1_ seedlings at six leaf stage were exposed to chilling stress at 10°C/7°C (day/night), 200 μmol m^-2^ s^-1^ PPFD for 21 days, while the other half were kept in the 25°C/20°C that served as a control. Except for the day/night temperature change, other growth conditions were the same for the chilled and control plants. The 5th leaves were sampled at day 21 after chilling stress for analysis of the content of H_2_O_2_, $O_{2}^{.-}$, lipid peroxidation, NO and GSNOR content, and the activities of NADP-dehydrogenases.

A pure culture of *C. fulvum* F5 was donated by Prof. Xu Tong, Zhejiang University (Hangzhou, China). Leaves from T_1_ transgenic tomato lines as well as control plants were inoculated with spore suspension (10^8^ per milliliter) of *C. fulvum*. The inoculated plants were kept in a moist container at 28°C/18°C, 95% relative humidity. Each treatment comprised 20 plants in replicates of three. The incidence and severity of tomato mold disease were measured 6 days post-pathogen inoculation. Disease severity was estimated using a Disease Index (DI) calculated from disease grades 0–5, using the following formula.

$$\text{DI}=\frac{\text{Sum of individual}\times \text{leaf ratings}}{\text{Maximum disease score}\times \text{Number of leaves sample}}\times 100$$

### Determination of H_2_O_2_, $O_{2}^{.-}$ and Lipid Peroxidation in Leaves

H_2_O_2_ content in leaves was determined spectrophotometrically by a peroxidase assay according to Willekens et al. (1997). The $O_{2}^{.-}$ contents was analyzed using the method of Elstner and Heupel (1976). The level of lipid peroxidation in leaves was determined by quantifying the malondialdehyde (MDA) equivalents using 2-thiobarbituric acid (TBA) as described by Hodges et al. (1999).

### Enzymatic and Non-enzymatic Antioxidants Determination in Ascorbate–Glutathione Cycle

The monodehydroascorbate reductase (MDHAR, EC 1.6.5.4) activity was measured using 1 U of ascorbate oxidase, and the oxidation rate of NADH was followed at 340 nm (Rao and Ormrod, 1995). The glutathione reductase (GR, EC 1.6.4.2) activity was measured according to Foyer and Halliwell (1976), as based on the rate of decrease in the absorbance of NADPH at 340 nm. The extraction and assay of reduced (GSH) and oxidized (GSSG) glutathione were performed as described by Rao and Ormrod (1995). The GSH concentration was obtained by subtracting the GSSG concentration from the total concentration. The levels of total ascorbate (AsA+DHA) and AsA were measured according to Law et al. (1983) with minor modification. The absorbance at 525 nm was read to determine reduced AsA according to the standard curve of known AsA concentrations. DHA was calculated as the difference between the total AsA and reduced AsA. L-galactono-1,4-lactone dehydrogenase (GaILDH) activity was determined according to the method of Tabata et al. (2001). γ-GCS was extracted and measured by the method of Shan and Liang (2010).

### Enzyme Activity Estimation

G6PDH (EC 1.1.1.49) activity was determined by following the reduction of NADPH according to Corpas et al. (2013). 6-phosphogluconate dehydrogenase (6PGDH, EC 1.1.1.44) activity was determined spectrophotometrically by recording the reduction in NADP at 340 nm (Valderrama et al., 2006). NADP–ICDH activity (EC 1.1.1.42) was measured by following the NADPH reduction according to Corpas et al. (2013). NADP-ME activity was assayed using a mixture of 50 mM Tris-HCl pH 7.5, 0.5 mM NADP, 10 mM malate, 10 mM MgCl_2_ and 10 μL of extract containing the enzyme (Badia et al., 2015).

### Determination of NO, SNO, and GSNO Content

NO was determined with hemoglobin assay following Pasqualini et al. (2009). Total SNO levels were determined by Saville’s method (Saville, 1958). Proteins were extracted in 100 mM Tris HCl, pH 6.8. The extracts were incubated for 5 min with an equivalent volume of solution A (1% sulfanilamide dissolved in 0.5 M HCl) in the presence or absence of solution B (solution A plus 0.2% HgCl_2_), allowing the development of the diazonium salt. The formation of the azo dye product was obtained by reacting the two samples for an additional 5 min with an equal volume of solution C [0.02% of *N*-(1-naphthyl) ethylenediamine dihydrochloride dissolved in 0.5 M HCl], and the absorbance was subsequently read at 550 nm with a spectrophotometer (Ultrospec 1100 pro, AmershamPharmacia Biotech). S-NOHCy was quantified as the difference of absorbance between solution B and A (B-A), comparing the values with a standard curve made from a solution of GSNO (Sigma–Aldrich). The results were normalized against whole cell-lysate protein content, measured by the method of Bradford (1976). The content of GSNO was determined according to Airaki et al. (2011) using liquid chromatography-electrospray/mass spectrometry (LC–ES/MS) method.

### Determination of Activities of *S*-Nitrosoglutathione (GSNOR), NOS, and NR

GSNO was purchased from Sigma (Sigma Aldrich, Shanghai, China). GSNOR activity was determined at 25°C in 0.1 M sodium phosphate, pH 7.5, by monitoring the consumption of NADH and GSNO (e_340_ = 7.06 mM^-1^ cm^-1^) in a Cary 400 Bio spectrophotometer. One unit of activity corresponds to 1 mol of coenzyme transformed per minute. The activity of NOS was determined by a NOS assay kit (Sigma–Aldrich).

The OD of NOS was monitored at 540 nm. Nitrate reductase (NR) activity was assayed spectrophotometrically as described by Lea et al. (2004).

### Western Blot Analysis for Tyrosine-Nitrated Proteins Detection

Western blot analysis for the detection of tyrosine-nitrated proteins was undertaken according Yadav et al. (2013). Briefly, after running SDS-PAGE, the gel was washed in transfer buffer [20% glycine, 5% tris (hydroxymethyl) aminomethane and 10% methanol, 4°C] for at least 15 min. The membrane was then incubated with a rabbit polyclonal antibody (anti-3NT antibody obtained from Sigma–Aldrich) against 3-nitrotyrosine in a dilution of 1:1000 in blocking buffer, for 2 h at room temperature on an orbital shaker. Thereafter, the membrane was washed in wash buffer three times for 5 min each and incubated in secondary antibody (antirabbit IgG conjugated to alkaline phosphatase antibody) for 1 h at RT on an orbital shaker. Both primary and secondary antibodies were obtained from Sigma–Aldrich.

### Statistical Analyses

All data presented are mean values of four repetitions of each treatment. Data were statistically analyzed using analysis of variance (AVONA), and tested for significant (*P* ≤ 0.05) treatment differences using Tukey’s test. Orgin pro 7.5 version was used to prepare graphs.

## Results

### Production of Transgenic Tomato Plants by Overexpressing *AdBiL* Gene

**FIGURE 1:**
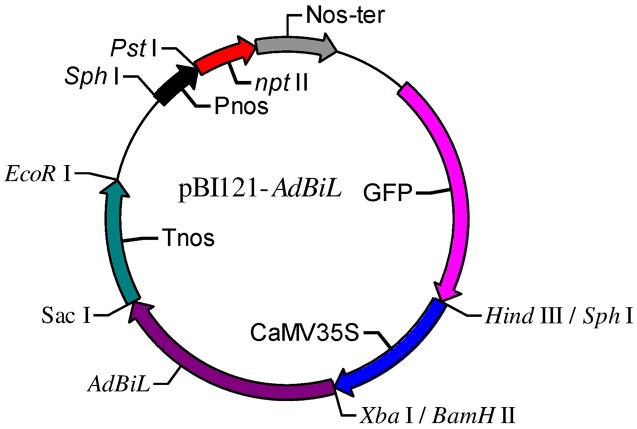
Schematic diagram of the T-DNA region of the construct pBI121- *AdBiL*. Tnos: nopaline synthase terminator; Pnos: nopaline synthase gene promoter; CaMV35S: cauliflower mosaic virus 35S promoter.

**FIGURE 2:**
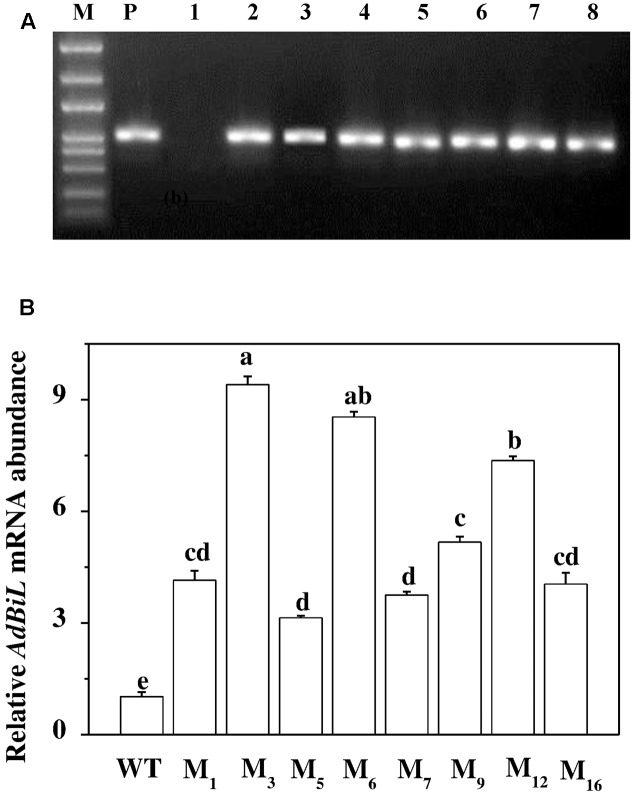
Detection of *AdBiL* gene in transgenic tomato. **(A)** PCR analysis. M: DNA ladder Marker; lane P: plasmids as positive control; lane 1: untransformed plants (WT); lanes 2–8: independent transgenic lines. **(B)** Real-time PCR analysis. cDNA from T_1_ transformed lines (M_1_, M_3_, M_5_, M_6_, M_7_, M_9_, M_12_, M_16_). Means followed by the same letter are not significantly different according to Tukey’s test (*P* < 0.05).

Transgenic tomato plants expressing *AdBiL* were generated by using the *Agrobacterium*-mediated transformation technique adopted for tomato plants. A binary vector carrying cauliflower mosaic virus (CaMV) 35S promoter and *AdBiL* cDNA was constructed and used for transformation (**Figure 1**). The integration of the *AdBiL* gene in putative transgenic plants (T_0_) was initially confirmed by PCR analysis using total genomic DNA isolated from leaves of transformed and untransformed plants with primers designed to amplify partial sequence of *AdBiL* gene. Seven T_0_ transgenic lines were used for PCR analysis with one untransformed line as negative control. An expected 751 bp fragment was observed with the *AdBiL* gene specific primers, whereas no corresponding band was detected in the untransformed plants (**Figure 2A**). Expression of the *AdBiL* gene was confirmed by quantitative real time PCR (qPCR) analysis. The transcript expression levels of *AdBiL* gene in the transgenic lines were higher than that in the untransformed plant (**Figure 2B**).

### Overexpression of *AdBiL* Gene Improved Tolerance to Chilling Stress in Tomato

To study whether overexpression of *AdBiL* affected tomato tolerance to chilling temperature, growth parameters such as fresh and dry weights of root and shoot as well as the ratio of root to shoot were analyzed. When plants grown at 25°C/20°C (day/night), no distinct differences were observed between the growth of the transgenic lines (M_3_, M_6_ and M_12_) and non-transformed plants. However, following cultivation of plants at 10°C/8°C for 21 days, severe symptoms of chilling injury were noticed in untransformed plants, while transgenic plants showed only mild symptoms of chilling injury. The fresh weight and dry weight of root and shoot as well as total biomass production were all distinctly higher in T_1_ transgenic plants compared with untransformed plants under chilling stress (**Table 1**). Similarly, chlorophyll contents in three transgenic lines such as M_3_, M_6_, and M_12_ were 44.28, 31.60, and 23.8% higher than that of untransformed plants, respectively. These results indicate that overexpression of *AdBiL* conferred chilling tolerance to tomato plants.

**Table 1:** Effects of chilling stress on biomass accumulation and chlorophyll content in tomato plants as influenced by *AdBiL* overexpression.

Treatment	Fresh weight			Dry weight			Chlorophyll (mg g^-1^ FW)
			
	Root(g)	Shoot (g)	Root–shoot ratio	Root(g)	Shoot (g)	Root–shoot ratio	
Control	1.322 c	14.725 c	0.090 c	0.171 d	1.154 c	0.171 d	15.38 c
M_3_	2.512 a	21.176 a	0.119 a	0.374 a	2.012 a	0.374 a	22.19 a
M_6_	2.194 b	19.064 ab	0.115 ab	0.322 b	1.865 b	0.322 b	20.24 b
M_12_	1.961 b	17.377 b	0.113 b	0.266 c	1.782 b	0.266 c	18.93 b


*Data are the means of four replicates. Means followed by the same letter are not significantly different according to Tukey’s test (*P* < 0.05). WT: untransformed plants; M_3_, M_6_, M_12_: lines of T_l_ transgenic plants.*

### Overexpression of *AdBiL* Alleviated Chilling-Induced Oxidative Stress

**FIGURE 3:**
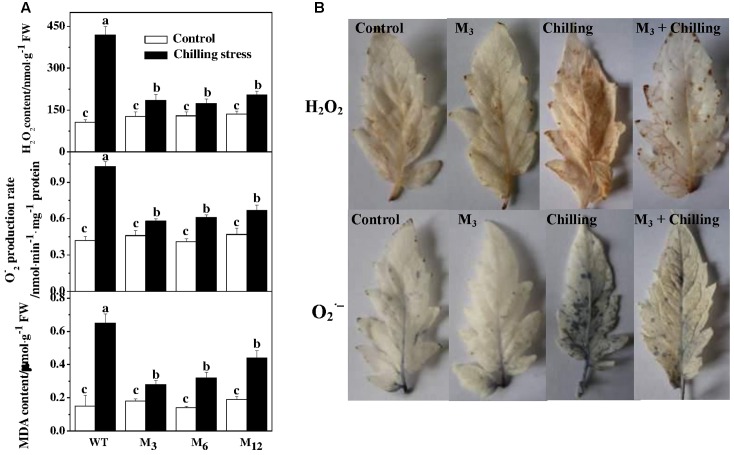
Effects of *AdBiL* overexpression on ROS accumulation and MDA content in leaves of tomato plants under chilling stress. **(A)** Quantitative measurements of H_2_O_2_, $O_{2}^{-}$ production and MDA level. **(B)** Visualization of H_2_O_2_ and O_2_^-^ accumulation by DAB and NBT staining, respectively. One half of untransformed and T_1_ seedlings at six leaf stage were exposed to chilling stress at 10°C/7°C (day/night) for 21 days, while the other half were kept at 25°C/20°C that served as a control. Data are the means of four replicates with SD shown by vertical bars. Means followed by the same letter are not significantly different according to Tukey’s test (*P* < 0.05). WT: untransformed plants; M_3_, M_6_, M_12_: lines of T_l_ transgenic lines.

To determine a potential involvement of ROS in chilling-induced growth inhibition, we attempted to quantify the accumulation of H_2_O_2_ and $O_{2}^{.-}$ in the leaves of *AdBiL* overexpression plants and the control. Notably, overexpression of *AdBiL* did not affect ROS accumulation under normal conditions. However, a dramatic increase in H_2_O_2_ content was observed in the leaves under chilling stress, more obviously in untransformed plants, which was attenuated in the leaves of *AdBiL* overexpressing plants subjected to chilling stress (**Figure 3A**). Furthermore, we attempted to detect *in situ* accumulation of H_2_O_2_ and $O_{2}^{.-}$ by using 3,3′-diaminobenzidine (DAB) and NBT staining procedures, respectively (**Figure 3B**). Consistent with quantitative results, a noticeable increase in H_2_O_2_ and $O_{2}^{.-}$ accumulation was observed in the leaves of untransformed plants compared to that in transformed plants (M_3_). To further clarify potential involvement of excessive ROS accumulation in membrane lipid peroxidation, we quantified MDA content in leaves. Similar to ROS accumulation, chilling stress significantly increased MDA content in leaves, suggesting that chilling stress induced oxidative stress in tomato plants. However, lipid peroxidation under chilling stress was strongly attenuated by overexpression of *AdBiL*, indicating that overexpression of *AdBiL* significantly alleviated chilling-induced oxidative stress (**Figure 3A**).

### Effects of *AdBiL* Overexpression on Content of NO, SNOs, GSNO and Activities of NOS and GSNOR under Chilling Stress

**FIGURE 4:**
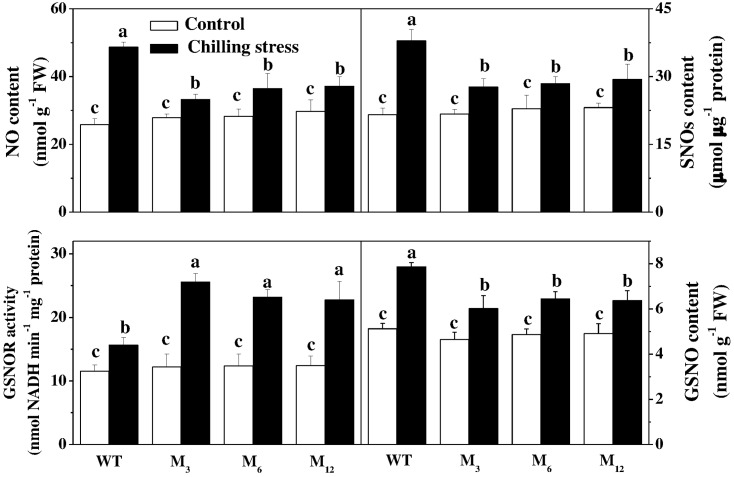
Effects of *AdBiL* overexpression on the content of NO, SNOs, GSNO, and GSNOR activity in leaves of tomato plant under chilling stress. Data are the means of four replicates with SD shown by vertical bars. Means followed by the same letter are not significantly different according to Tukey’s test (*P* < 0.05). WT: untransformed plants; M_3_, M_6_, M_12_: lines of T_l_ transgenic lines.

To determine a possible role of RNS metabolism in *AdBiL* overexpression plants under chilling stress, we attempted to quantify the accumulation of NO, SNOs and activities of NOS, GSNOR in the leaves. No noticeable differences in NO, SNOs, and GSNO were observed in the leaves between untransformed and transgenic plants under normal growth conditions. However, a significant increase in NO, SNOs, and GSNO was detected in the leaves under chilling stress, while the contents of NO, SNOs, and GSNO decreased significantly in the leaves of *AdBiL* gene overexpressing plants subjected to chilling stress compared with the untransformed plants. The declines of NO, SNOs, and GSNO were 31.72, 26.92, and 23.54%, respectively in the leaves of M_3_ line while 23.62, 22.65, and 18.96% in the leaves of M_12_ line relative to untransformed control (**Figure 4**). Furthermore, *AdBiL* overexpression also induced the activity of *S*-nitrosoglutathione reductase (GSNOR) during chilling stress, while inhibited the activity of nitric oxide synthase (NOS) and NR (**Table 2**). These results provide strong evidence that *AdBiL* gene plays critical role in maintaining the balance between GSNOR, NR and NOS under chilling-induced oxidative stress.

**Table 2:** Effects of *AdBiL* overexpression on enzyme activities related with AsA–GSH cycle in leaves of tomato plant under chilling stress.

Treatment	GR activity (μmol⋅min^-1^ mg^-1^ protein)	MDHAR activity (μmol⋅min^-1^ mg^-1^ protein)	G6PDH activity (nmol⋅min^-1^ mg^-1^⋅protein)	6PGDH activity (nmol⋅min^-1^ mg^-1^⋅protein)	NADP-ICDH activity (nmol⋅min^-1^ mg^-1^⋅protein)	NADP-ME activity (nmol⋅min^-1^ mg^-1^⋅protein)	NOS activity (U mg^-1^ protein)	NR activity μmol NO_2_ h^-1^g^-1^ FW
Control	0.071 c	0.238 c	31.83 c	18.91 c	30.15 c	20.16 c	0.67 a	1.42 a
M_3_	0.142 a	0.561 a	47.06 a	44.66 a	44.82 a	37.84 a	0.42 c	0.64 b
M_6_	0.115 b	0.497 a	37.27 b	41.18 a	37.26 ab	29.76 b	0.48 bc	0.71 b
M_12_	0.108 b	0.354 b	35.95 b	32.74 b	32.84 b	27.37 b	0.53 b	0.75 b


*Data are the means of four replicates. Means followed by the same letter are not significantly different according to Tukey’s test (*P* < 0.05). WT: untransformed plants; M_3_, M_6_, M_12_: lines of T_l_ transgenic plants.*

### Effects of *AdBiL* Overexpression on the Content of AsA, GSH and Activity of GaILDH in Leaves of Tomato Plant under Chilling Stress

**FIGURE 5:**
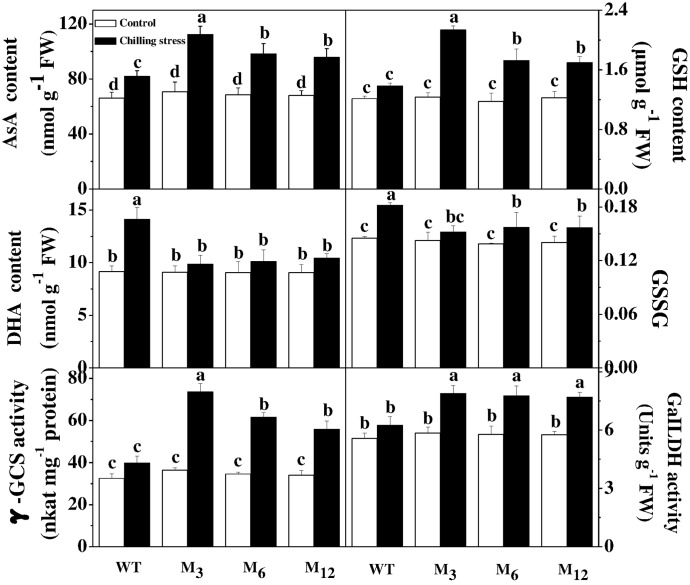
Effects of *AdBiL* overexpression on the content of AsA, GSH and the activity of GaILDH in leaves of tomato plant under chilling stress. Data are the means of four replicates with SD shown by vertical bars. Means followed by the same letter are not significantly different according to Tukey’s test (*P* < 0.05). WT: untransformed plants; M_3_, M_6_: lines of T_l_ transgenic lines.

To determine the involvement of altered cellular redox status in response to *AdBiL* gene overexpression, we examined the redox state of AsA and GSH pool (**Figure 5**). The content of AsA, GSH, the ratio of AsA/DHA and GSH/GSSG were all increased in the leaves of transgenic plants under chilling stress, accounting for 37.10, 54.61, 96.47, and 24.95% higher in M_3_ line, respectively. The activity of GalLDH was increased by 25.96% in the leaves of transgenic plants under chilling stress. Furthermore, activity of γ-GCS, the key enzyme for glutathione biosynthesis, was also increased by 89.63% in leaves of transgenic M_3_ line under chilling stress, relative to that of untransformed control. These results strongly suggest that *AdBiL* gene is involved in the cellular redox homeostasis under chilling stress.

### Effects of *AdBiL* Overexpression on Enzyme Activities Related to AsA–GSH Cycle in Leaves of Tomato Plant under Chilling Stress

Next, we examined the activities of enzymes associated with the AsA–GSH cycle in leaves of tomato plants under chilling stress (**Table 2**). We found that the transgenic lines showed increased activities of enzymes involved in the AsA–GSH cycle compared with that in untransformed control under low temperature conditions (**Table 2**). The enzyme activities of GR, MDHAR, G6PDH, 6PGDH, NADP-ICDH, and NADP-ME were all increased in the leaves of transgenic plants under chilling stress, which were increased by 37.10, 19.15, 96.47, and 24.95% in M_3_ line relative to untransformed control, respectively. These results further confirm that a high level of *AdBiL* transcript facilitates higher enzyme activities related to the AsA–GSH cycle under chilling stress. Such enhancement in the activity of those enzymes through overexpression of *AdBiL* resulted in an increased biosynthesis of AsA and GSH and thus *AdBiL* overexpressing plants suffered less under chilling stress, as evidenced by the lower accumulation of ROS, SNOs and MDA.

### Overexpression of *AdBiL* Confers Tomato Tolerance to Fungal Infections

**FIGURE 6:**
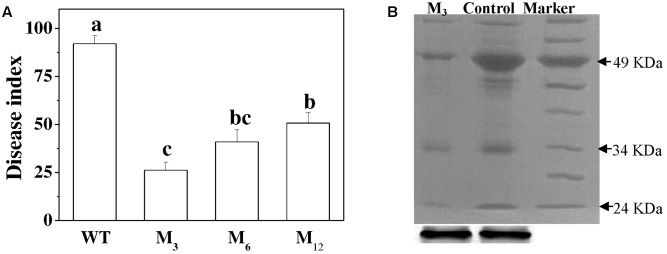
Evaluation of *AdBiL* overexpression plants against *Cladosporium fulvum* infections. **(A)** Disease index (DI) determination. Data are the means of four replicates with SD shown by vertical bars. Means followed by the same letter are not significantly different according to Tukey’s test (*P* < 0.05). WT: untransformed plants; M_3_, M_6_, M_12_: lines of T_l_ transgenic plants. Means followed by the same letter are not significantly different according to Tukey’s test (*P* < 0.05). **(B)** Western blot analyses of anti-nitrotyrosine labeled proteins.

**FIGURE 7:**
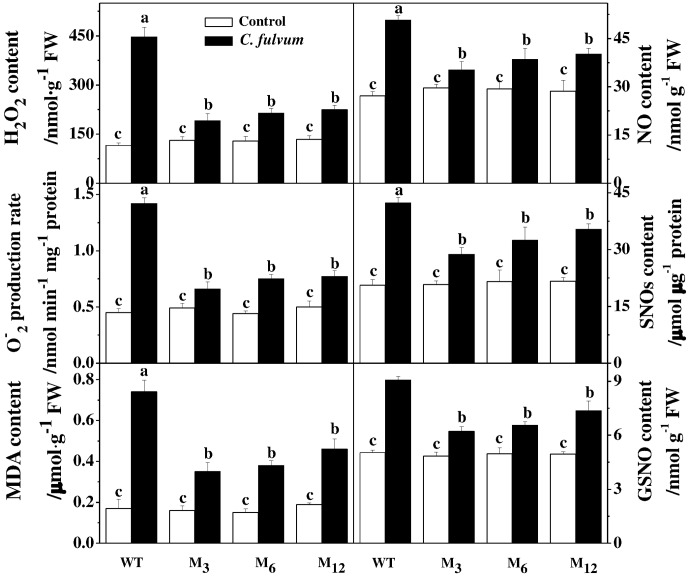
Effects of *AdBiL* overexpression on the content of ROS, NO, SNOs, and GSNO in leaves of tomato plant infected with *C. fulvum.* Data are the means of four replicates with SD shown by vertical bars. Means followed by the same letter are not significantly different according to Tukey’s test (*P* < 0.05). WT: untransformed plants; M_3_, M_6_, M_12_: lines of T_l_ transgenic lines.

To assess whether overexpression of the *AdBiL* gene could affect tolerance of tomato plants to biotic stress, we challenged both transformed plants and the control plants with the fungal pathogens, *C. fulvum*. Analysis of DI revealed that the control plants showed severe disease symptoms after infection, which were greatly attenuated in the transgenic plants. For instance, DI in the infected untransformed tomato plant leaves was approximately 92.04%, which was significantly higher than that of transgenic M_3_ plants showing a DI of 26.18% (**Figure 6A**). Moreover, compared with transformed plants, tyrosine nitration protein in 49KD, 34KD, and 24KD of control plants was greatly stimulated after inoculation with *C. fulvum*, causal agent of leaf mold disease (**Figure 6B**). Additionally, no statistically significant change was observed in ROS accumulation and the content of MDA, NO, SNOs, and GSNO between transgenic lines and control plants under normal conditions. However, inoculation with *C. fulvum* strongly increased the content of H_2_O_2_, $O_{2}^{.-}$, MDA, NO, SNOs, and GSNO in the leaves of all genotypes, more obviously in the untransformed plants (**Figure 7**).

## Discussion

In recent years, plant RING E3 ligases have received much attention by the plant scientists due to their putative involvement in diverse defense responses to abiotic and biotic stresses. In particular, plant RING E3 ligases impart broad-spectrum resistance to *Alternaria alternata* (Kabbage et al., 2010), salt stress (Kabbage et al., 2010), drought stress (Li et al., 2016) and *Heterodera schachtii* infection (Hewezi et al., 2016). They also play a key role in ABA and ethylene signaling by regulating ABA-mediated seed germination and cell death, and ethylene-mediated fruit ripening. Despite a keen interest in plant RING motif-harboring ubiquitin E3 ligases for their involvement in diverse cellular and developmental processes, a few reports on the functional correlation between RING E3 ligases and ROS or NO signal-mediated stress responses are currently available. In the present study, we demonstrated potential roles of *AdBiL* gene in mediating response of tomato plants to chilling stress and fungal infection. Our results revealed that *AdBiL* gene plays an essential role in maintaining the balance in the activity of key enzymes involved in AsA–GSH cycle and redox homeostasis during chilling stress and tomato leaf mold disease, resulting in a lower accumulation of ROS and RNS as well as an attenuated oxidative stress.

Two main enzymatic pathways for NO production in plants have been reported. The first pathway involves NR which catalyzes the reduction of nitrite to NO. NR is a main NO source during abiotic stress and in the signaling pathway that is triggered by abscisic acid leading to stomatal closure (Chamizo-Ampudia et al., 2017). The second one involves a putative NOS. NOS catalyzes the NADPH- and O_2_-dependent oxidation of L-Arg to citrulline and NO, and utilizes FAD, FMN and tetrahydrobiopterin as redox cofactors (Besson-Bard et al., 2009; Niu and Liao, 2016). Previous studies have revealed that GSNO reductase (GSNOR) is evolutionarily conserved from bacteria to plant, and is critical for cellular NO and SNO homeostasis (Liu et al., 2001; Niu and Liao, 2016). GSNOR plays a crucial role in minimizing nitrosative stress manifested by changes in GSNO levels in plants. The expression of GSNOR is significantly induced by NaCl treatment, whereas Put and Spm also stimulate GSNOR under salinity (Tanou et al., 2014). In the present study, we showed that chilling stress caused remarkable increases in NO and SNOs accumulation, disturbed ROS-RNS balance and resulted in nitrosative stress, which was in accordance with previous studies in *Baccaurea ramiflora* (Bai et al., 2012) and poplar (Cheng et al., 2015), but in contrast with the results observed in citrus (Ziogas et al., 2013), which demonstrated that the gene expression and enzymatic activity of GSNOR of citrus plants displayed significant changes in response to adverse environmental conditions, particularly cold stress. GSNOR patterns are enhanced by heat, cold or drought but are suppressed by dark or salinity (Ziogas et al., 2013). However, in the current study, overexpression of *AdBiL* gene inhibited NO over accumulation via regulation of NOS-like and NR enzymatic pathways in the transgenic plants.

Ascorbate (AsA) and glutathione (GSH) play important roles in plant response to abiotic and biotic stresses by providing a more efficient enzymatic mechanism of H_2_O_2_ breakdown than that of catalase and peroxidase (Xing et al., 2016). GR, MDHAR, and DHAR are considered as the key enzymes in antioxidant defense systems that play vital role in GSH regeneration in AsA–GSH cycle, whereas NADPH is required to maintain an adequate intracellular pool of GSH that acts as main reductant against ROS in the plant cells (Zou et al., 2016). In addition, MDHAR is responsible for the reduction of MDA to AsA and increases reduced form of AsA (Park et al., 2016). The current study showed that the activities of GR and MDHAR were distinctly increased by *AdBiL* gene overexpression, and the increase was accompanied by a simultaneous reduction in ROS and RNS (**Figures 3**, **4**). This finding was in good agreement with an earlier study that showed that the RING finger E3 ligase *STRF1* was involved in the tolerance of *Arabidopsis thaliana* to salt, ionic and osmotic stresses and a reduction in ROS accumulation during salt stress (Tian et al., 2015). We also found that the key regulatory enzymes of pentose phosphate pathway (PPP), G6PDH, 6PGDH, NADP-ICDH and NADP-ME, were all increased in the leaves of transgenic plants under chilling stress. Similar trend has been shown in leaves of pepper plants after exposure of plant to low temperatures (Airaki et al., 2012). In non-photosynthetic cells, the PPP is a major source of reductant (i.e., NADPH) for maintaining the redox potential necessary to protect against oxidative stress (Kruger and von Schaewen, 2003). G6PDH played a central role in the control of output of GSH from its oxidized form (GSSG) by utilizing NADPH (Wang et al., 2008). NADPH participates in reducing oxidized glutathione (GSSG) content to keep reduced glutathione and thioredoxin levels. GSSG can be recycled to GSH by GR coupled with NADPH as an electron donor (Jimenez-Quesada et al., 2016). Thus, cellular redox status is maintained by the GSH/GSSG ratio, however, an oxidative stress results in decreased GSH/GSSG. NADPH could provide cells with large amounts of ATP, much of which is required for the activity of NADPH oxidase and thioredoxin reductase (Segal, 2016). H_2_O_2_ also participated in stimulating GR activity under salt stress and the components of ascorbate–glutathione cycle (cytosolic glutaredoxin and MDAR) (Davletova et al., 2005), which finally result in the enhanced glutathione cycling rate and thus the increased GSH levels under chilling stress. Therefore, we propose that the activation of key enzymes in AsA–GSH cycle and PPP pathways might be one of the mechanisms involved in ROS and RNS scavenging, thereby reducing oxidative- and nitrosative stress-induced damaged to cell membrane in tomato plants.

While ascorbate and glutathione play critical roles in signal regulation and/or transmission during cell death and defense responses, it is less clear how their functions in controlling growth are associated with the regulation of ROS and RNS metabolism. GSH could not only eliminate active oxygen species as substrates through Halliwell-Asada pathway, it also could synthetize *S*-nitrosoglutathione (GSNO) through the catalysis of NOS with NO, interacting with its derivative reactive nitrogen in a process called *S*-nitrosylation. GSNO has been reported to function as a mobile reservoir of NO bioactivity and can mediate the signaling pathway throughout specific post-translational modification of redox-sensitive proteins by a reaction of *trans-*nitrosylation from GSNO to Cys-NO (Airaki et al., 2011).

GSNO is mainly involved in the transfer of NO to other thiol molecules in the cell *in vivo*, thus forming stable SNOs. GSNOR plays a dual role in regulating NO, GSNO, Protein-SNO and SNOs in dynamic level. The first role is to terminate NO signaling and make SNOs level including L-GSNO down to normal level. The enzyme GSNOR can decompose GSNO, however, mechanical injury down regulates its level to a different degree (gene and protein expression as well as its specific activity) in hypocotyls of sunflower (Chaki et al., 2011). In pea leaves, after mechanical wounding, an increased GSNOR activity and content of SNOs has been reported, accompanied by an increase in NOS activity (Corpas et al., 2008). Nevertheless, in wild-type *Arabidopsis* exposed to heat stress, GSNOR protein expression was similar in both control and heat-stressed wild-type leaves. Nitrosylation mediated by GSNOR enzyme can regulate proteins activities including mitochondrial aldehyde dehydrogenase protein, protein disulfide isomerase, heat shock protein Hsp60 and raffinose kinase inhibitor protein (Lopez-Sanchez et al., 2008). The high dose disturbance of SNOs homeostasis induced by NO and protein nitrosylation can be reversed by GSNOR enzyme. Due to mere chemical transnitrosylation reactions, indeed, the redox couples GSH/GSNO and protein-SH/protein-SNOs are in a dynamic equilibrium therefore, by directly reducing GSNO, GSNOR indirectly decreases the concentration of protein SNOs (Giacomo et al., 2012). In our study, distinct decrease of the content of ROS ($O_{2}^{-}$ and H_2_O_2_), reactive nitrogen and GSNO accumulation was observed in overexpressing lines as compared with untransformed plants under chilling stress. The results were consistent with the previous studies in pea (Corpas et al., 2008), *Arabidopsis* (Garcês et al., 2001) and Pelargonium (Arasimowicz et al., 2009) but in contrast to the report in sunflower (Chaki et al., 2011), which showed no direct correlation between oxidative stress and NO generation. In contrast, the content of AsA, GSH and activities of GILDH, γ-GCS and GSNOR were significantly increased (**Figure 5** and **Table 2**). The results revealed that *AdBiL* gene could modulate the content of NO, SNOs in transgenic plants by increasing biosynthesis of AsA and GSH and GSNOR activity. *S*- nitrosylation mediated by GSH and GSNOR was involved in the cross mechanisms of plant resistance to abiotic and biotic stresses such as chilling stress and leaf mold disease by minimizing ROS and NO production. *S*-nitrosylation has emerged as a novel regulator in cell signaling and response to stress conditions (Lázaro et al., 2013). Here we showed tyrosine nitration protein was activated in the plants infected with leaf mold disease, while the inhibition could be recovered in *AdBiL* gene overexpressing lines. These results were in line with the previous studies which showed that mechanical wounding induced a nitrosative stress by down-regulation of GSNO reductase and an increase in *S*-nitrosothiols in sunflower seedlings (Chaki et al., 2011). However, there are also contrasting reports that the increase in NO^⋅^ content under salt conditions was not related to an enhanced mitochondrial protein *S*-nitrosylation (Camejo et al., 2013).

In summary, we demonstrated that *AdBiL* gene had an essential role in maintaining cellular ROS and RNS homeostasis by modulating GSH, AsA and GSNOR simultaneously. Furthermore, less accumulation of H_2_O_2_ and $O_{2}^{.-}$ coupled with lower NO and SNOs were observed in *AdBiL* gene overexpressing lines relative to untransformed plants under chilling stress. This implies a possible physiological role of *AdBiL* in the activation of the key enzymes of AsA–GSH cycle, PPP and down-regulation of GSNO reductase, thereby reducing oxidative and nitrosative stress. Taken together, these results highlight a protective role of *AdBiL* gene which may have potential implication in developing chilling-tolerant and leaf mold disease-resistant crop varieties through genetic manipulation.

## Author Contributions

AL and SC conceived and designed the research; SC, HZ, MW, JL, and FW performed the experiments and analyzed the data; ZW supervised the study; SC, GA, and AL wrote the manuscript. All authors read and approved the manuscript.

## Conflict of Interest Statement

The authors declare that the research was conducted in the absence of any commercial or financial relationships that could be construed as a potential conflict of interest.
